# Supplementary material for: A Likelihood Approach for Real-Time Calibration of Stochastic Compartmental Epidemic Models
Source: PLoS Comput Biol. 2017 Jan 17;13(1):e1005257. doi: 10.1371/journal.pcbi.1005257 (PMC5240920; doi:10.1371/journal.pcbi.1005257)
Supplement: S1 File — (TAR.GZ) [file pcbi.1005257.s014.tar.gz › HSPH_Online-SI-Revision/PF08/n100-severe/n100-severe_PF2_table8.pdf]

1	newly 3 weeks spec	405.	1651.29	{1596., 1700.}	307.726	0.610224	newly 3 weeks cumul	1885.	6191.53	{6094., 6283.}	228.463	0.516456	newly 5 weeks spec	212.	1077.55	{1030., 1118.}	408.278	0.705866	newly 5 weeks cumul	2436.	8602.47	{8476., 8722.}	253.139	0.547914	newly 42 weeks cumul	2940.	12 839.1	{12 516., 13 102.}	336.703	0.64012
2	newly 3 weeks spec	32.	1091.35	{1051., 1131.}	3310.47	1.53263	newly 3 weeks cumul	144.	4462.87	{4413., 4520.}	2999.22	1.49123	newly 5 weeks spec	7.	613.73	{584., 644.}	8667.57	1.94258	newly 5 weeks cumul	163.	5895.37	{5844., 5947.}	3516.79	1.55831	newly 49 weeks cumul	163.	7715.44	{7653., 7768.}	4633.4	1.67517
3	newly 3 weeks spec	20.	1204.14	{1163., 1242.}	5920.7	1.7795	newly 3 weeks cumul	87.	4928.02	{4864., 4984.}	5564.39	1.75313	newly 5 weeks spec	9.	673.65	{640., 707.}	7385.	1.87387	newly 5 weeks cumul	101.	6499.23	{6440., 6555.}	6334.88	1.80853	newly 50 weeks cumul	112.	8495.44	{8420., 8554.}	7485.21	1.87996
4	newly 3 weeks spec	173.	1102.92	{1063., 1140.}	537.526	0.804321	newly 3 weeks cumul	880.	4477.21	{4398., 4540.}	408.774	0.706467	newly 5 weeks spec	105.	623.65	{589., 663.}	493.952	0.773301	newly 5 weeks cumul	1112.	5930.45	{5844., 6010.}	433.314	0.726926	newly 48 weeks cumul	1341.	7802.63	{7677., 7939.}	481.852	0.764734
5	newly 3 weeks spec	780.	1393.38	{1322., 1462.}	78.6385	0.251626	newly 3 weeks cumul	2856.	5251.11	{5077., 5408.}	83.8624	0.264326	newly 5 weeks spec	402.	900.77	{843., 961.}	124.072	0.349808	newly 5 weeks cumul	3744.	7276.04	{7028., 7524.}	94.3387	0.288378	newly 42 weeks cumul	4829.	10 789.9	{10 358., 11 203.}	123.44	0.348905
6	newly 3 weeks spec	252.	1375.81	{1327., 1433.}	445.956	0.736958	newly 3 weeks cumul	1205.	5497.98	{5405., 5607.}	356.264	0.659165	newly 5 weeks spec	97.	793.08	{758., 832.}	717.608	0.912271	newly 5 weeks cumul	1441.	7337.21	{7240., 7447.}	409.175	0.70683	newly 48 weeks cumul	1575.	9838.35	{9698., 10 046.}	524.657	0.795589
7	newly 3 weeks spec	63.	1228.74	{1188., 1268.}	1850.38	1.28999	newly 3 weeks cumul	435.	5026.52	{4955., 5082.}	1055.52	1.06276	newly 5 weeks spec	20.	697.39	{664., 726.}	3386.95	1.54217	newly 5 weeks cumul	497.	6653.11	{6583., 6712.}	1238.65	1.12666	newly 49 weeks cumul	509.	8727.2	{8644., 8797.}	1614.58	1.23415
8	newly 3 weeks spec	292.	1495.99	{1449., 1541.}	412.325	0.709431	newly 3 weeks cumul	1253.	5824.87	{5754., 5895.}	364.874	0.667317	newly 5 weeks spec	140.	902.93	{859., 948.}	544.95	0.80925	newly 5 weeks cumul	1606.	7890.61	{7805., 7974.}	391.321	0.69135	newly 45 weeks cumul	1911.	10 942.3	{10 753., 11 102.}	472.595	0.757816
9	newly 3 weeks spec	0.1	1866.07	{1815., 1920.}	1.86597×10^{6}	4.27082	newly 3 weeks cumul	4.	7530.04	{7458., 7593.}	188 151.	3.27473	newly 5 weeks spec	0.1	1060.49	{1017., 1102.}	1.06039×10^{6}	4.02526	newly 5 weeks cumul	4.	10 005.8	{9944., 10 074.}	250 044.	3.39818	newly 50 weeks cumul	4.	13 214.8	{13 143., 13 290.}	330 269.	3.519
10	newly 3 weeks spec	115.	2799.99	{2730., 2867.}	2334.77	1.38639	newly 3 weeks cumul	507.	9804.15	{9719., 9883.}	1833.76	1.28639	newly 5 weeks spec	26.	2008.12	{1951., 2056.}	7623.54	1.8877	newly 5 weeks cumul	597.	14 188.4	{14 060., 14 303.}	2276.62	1.37595	newly 43 weeks cumul	631.	23 687.9	{23 376., 23 967.}	3654.02	1.57448
11	newly 3 weeks spec	53.	1163.73	{1126., 1198.}	2095.72	1.34143	newly 3 weeks cumul	267.	4778.18	{4720., 4839.}	1689.58	1.25273	newly 5 weeks spec	7.	657.72	{620., 683.}	9296.	1.9726	newly 5 weeks cumul	288.	6310.62	{6255., 6375.}	2091.19	1.34067	newly 49 weeks cumul	300.	8244.82	{8185., 8299.}	2648.27	1.43905
12	newly 3 weeks spec	467.	2306.11	{2242., 2364.}	393.814	0.693489	newly 3 weeks cumul	2017.	8253.59	{8172., 8337.}	309.201	0.611924	newly 5 weeks spec	256.	1604.77	{1545., 1673.}	526.863	0.796972	newly 5 weeks cumul	2654.	11 781.9	{11 678., 11 900.}	343.928	0.647303	newly 42 weeks cumul	3292.	19 007.6	{18 770., 19 254.}	477.387	0.761447
13	newly 3 weeks spec	476.	1490.04	{1434., 1543.}	213.034	0.495401	newly 3 weeks cumul	2172.	5600.28	{5475., 5737.}	157.84	0.411279	newly 5 weeks spec	224.	965.11	{913., 1010.}	330.853	0.633986	newly 5 weeks cumul	2758.	7769.29	{7597., 7984.}	181.7	0.449709	newly 42 weeks cumul	3330.	11 563.	{11 263., 11 917.}	247.238	0.540507
14	newly 3 weeks spec	744.	1875.86	{1813., 1936.}	152.132	0.401471	newly 3 weeks cumul	3264.	6898.43	{6714., 7052.}	111.349	0.324935	newly 5 weeks spec	407.	1259.78	{1191., 1320.}	209.528	0.490371	newly 5 weeks cumul	4260.	9705.22	{9503., 9946.}	127.822	0.357522	newly 42 weeks cumul	5829.	14 957.5	{14 425., 15 412.}	156.604	0.40914
15	newly 3 weeks spec	170.	2202.49	{2139., 2255.}	1195.58	1.11238	newly 3 weeks cumul	891.	8088.89	{8008., 8180.}	807.844	0.957999	newly 5 weeks spec	60.	1473.26	{1419., 1534.}	2355.43	1.38994	newly 5 weeks cumul	1068.	11 375.2	{11 272., 11 473.}	965.096	1.02738	newly 44 weeks cumul	1164.	17 474.8	{17 270., 17 667.}	1401.27	1.17644
16	newly 3 weeks spec	648.	2118.66	{2069., 2173.}	226.954	0.514394	newly 3 weeks cumul	2744.	7782.89	{7663., 7938.}	183.633	0.452719	newly 5 weeks spec	394.	1411.21	{1357., 1466.}	258.175	0.55389	newly 5 weeks cumul	3600.	10 930.3	{10 793., 11 070.}	203.62	0.48231	newly 43 weeks cumul	4481.	16 766.4	{16 509., 16 998.}	274.166	0.573037
17	newly 3 weeks spec	33.	1412.57	{1367., 1457.}	4180.52	1.63135	newly 3 weeks cumul	309.	5723.48	{5666., 5774.}	1752.26	1.26769	newly 5 weeks spec	37.	801.96	{763., 837.}	2067.46	1.33569	newly 5 weeks cumul	396.	7588.7	{7544., 7640.}	1816.34	1.28247	newly 49 weeks cumul	405.	10 001.1	{9926., 10 072.}	2369.4	1.39259
18	newly 3 weeks spec	122.	1216.75	{1164., 1270.}	897.336	0.998593	newly 3 weeks cumul	687.	5016.59	{4934., 5091.}	630.217	0.863422	newly 5 weeks spec	37.	668.25	{626., 711.}	1706.08	1.25624	newly 5 weeks cumul	830.	6591.16	{6463., 6687.}	694.116	0.899849	newly 47 weeks cumul	928.	8470.44	{8269., 8634.}	812.763	0.960297
19	newly 3 weeks spec	77.	1041.6	{1002., 1078.}	1252.73	1.13102	newly 3 weeks cumul	381.	4215.62	{4153., 4275.}	1006.46	1.04391	newly 5 weeks spec	32.	597.28	{569., 631.}	1766.5	1.27067	newly 5 weeks cumul	445.	5603.6	{5547., 5659.}	1159.24	1.10009	newly 47 weeks cumul	467.	7444.41	{7367., 7522.}	1494.09	1.2025
20	newly 3 weeks spec	99.	1982.4	{1924., 2032.}	1902.42	1.30145	newly 3 weeks cumul	531.	7534.31	{7456., 7610.}	1318.89	1.15193	newly 5 weeks spec	19.	1255.53	{1208., 1310.}	6508.05	1.81984	newly 5 weeks cumul	601.	10 372.7	{10 281., 10 473.}	1625.91	1.23701	newly 45 weeks cumul	622.	15 009.9	{14 845., 15 182.}	2313.16	1.38257
21	newly 3 weeks spec	24.	1253.02	{1213., 1297.}	5120.92	1.71757	newly 3 weeks cumul	229.	5091.98	{5025., 5147.}	2123.57	1.34703	newly 5 weeks spec	14.	708.37	{674., 739.}	4959.79	1.70386	newly 5 weeks cumul	254.	6738.99	{6687., 6802.}	2553.15	1.42375	newly 49 weeks cumul	256.	8845.65	{8770., 8930.}	3355.33	1.53848
22	newly 3 weeks spec	57.	2202.52	{2146., 2274.}	3764.07	1.58692	newly 3 weeks cumul	329.	8515.92	{8427., 8589.}	2488.43	1.41302	newly 5 weeks spec	27.	1340.61	{1288., 1383.}	4865.22	1.69577	newly 5 weeks cumul	392.	11 580.2	{11 493., 11 695.}	2854.13	1.47042	newly 47 weeks cumul	426.	16 057.5	{15 897., 16 220.}	3669.38	1.57625
23	newly 3 weeks spec	0.1	1435.1	{1392., 1475.}	1.435×10^{6}	4.15676	newly 3 weeks cumul	12.	5886.86	{5826., 5949.}	48 957.2	2.69069	newly 5 weeks spec	0.1	814.82	{779., 845.}	814 720.	3.91087	newly 5 weeks cumul	12.	7781.29	{7723., 7826.}	64 744.1	2.81186	newly 51 weeks cumul	12.	10 175.8	{10 111., 10 240.}	84 698.	2.92838
24	newly 3 weeks spec	10.	1511.41	{1465., 1548.}	15 014.1	2.17927	newly 3 weeks cumul	103.	6187.8	{6118., 6256.}	5907.57	1.77868	newly 5 weeks spec	0.1	856.57	{826., 886.}	856 470.	3.93259	newly 5 weeks cumul	103.	8184.77	{8132., 8235.}	7846.38	1.90016	newly 52 weeks cumul	103.	10 723.5	{10 657., 10 781.}	10 311.2	2.0175
25	newly 3 weeks spec	0.1	1561.16	{1513., 1606.}	1.56106×10^{6}	4.19334	newly 3 weeks cumul	33.	6330.95	{6267., 6408.}	19 084.7	2.28294	newly 5 weeks spec	0.1	884.96	{846., 922.}	884 860.	3.9467	newly 5 weeks cumul	33.	8388.33	{8326., 8447.}	25 319.2	2.40515	newly 52 weeks cumul	33.	11 044.7	{10 972., 11 116.}	33 368.9	2.52464
26	newly 3 weeks spec	105.	1981.36	{1917., 2041.}	1787.01	1.27564	newly 3 weeks cumul	559.	7612.73	{7532., 7683.}	1261.85	1.13411	newly 5 weeks spec	34.	1213.66	{1169., 1270.}	3469.59	1.5524	newly 5 weeks cumul	657.	10 383.2	{10 295., 10 487.}	1480.4	1.19876	newly 46 weeks cumul	704.	14 590.6	{14 438., 14 757.}	1972.53	1.31648
27	newly 3 weeks spec	67.	1387.05	{1344., 1424.}	1970.22	1.31589	newly 3 weeks cumul	373.	5579.38	{5527., 5629.}	1395.81	1.17486	newly 5 weeks spec	19.	800.4	{767., 836.}	4112.63	1.62427	newly 5 weeks cumul	421.	7436.87	{7377., 7500.}	1666.48	1.2471	newly 47 weeks cumul	444.	9954.89	{9876., 10 035.}	2142.09	1.35064
28	newly 3 weeks spec	219.	1051.41	{988., 1112.}	380.096	0.680912	newly 3 weeks cumul	956.	4320.06	{4210., 4460.}	351.889	0.654915	newly 5 weeks spec	119.	588.49	{550., 626.}	394.529	0.693635	newly 5 weeks cumul	1227.	5696.58	{5517., 5911.}	364.269	0.66664	newly 46 weeks cumul	1619.	7393.09	{7136., 7691.}	356.645	0.659394
29	newly 3 weeks spec	122.	1058.63	{1018., 1098.}	767.73	0.938175	newly 3 weeks cumul	698.	4288.68	{4230., 4356.}	514.424	0.788437	newly 5 weeks spec	37.	598.59	{567., 630.}	1517.81	1.20856	newly 5 weeks cumul	790.	5687.54	{5607., 5770.}	619.942	0.857271	newly 47 weeks cumul	845.	7514.91	{7423., 7603.}	789.338	0.949041
30	newly 3 weeks spec	189.	2117.53	{2060., 2173.}	1020.39	1.04928	newly 3 weeks cumul	1013.	7715.35	{7637., 7809.}	661.634	0.88173	newly 5 weeks spec	86.	1428.59	{1388., 1470.}	1561.15	1.2203	newly 5 weeks cumul	1247.	10 890.2	{10 789., 10 992.}	773.308	0.941157	newly 43 weeks cumul	1455.	16 995.3	{16 769., 17 177.}	1068.06	1.06745
31	newly 3 weeks spec	1.	1525.73	{1484., 1565.}	152 473.	3.18337	newly 3 weeks cumul	19.	6217.01	{6162., 6272.}	32 621.1	2.51482	newly 5 weeks spec	0.1	868.86	{829., 909.}	868 760.	3.93873	newly 5 weeks cumul	19.	8233.73	{8178., 8291.}	43 235.4	2.63684	newly 52 weeks cumul	19.	10 811.4	{10 754., 10 875.}	56 801.9	2.75512
32	newly 3 weeks spec	28.	1448.84	{1408., 1488.}	5074.43	1.71376	newly 3 weeks cumul	154.	5894.69	{5817., 5960.}	3727.72	1.58292	newly 5 weeks spec	6.	818.97	{776., 856.}	13 549.5	2.13486	newly 5 weeks cumul	176.	7804.06	{7748., 7862.}	4334.13	1.6468	newly 50 weeks cumul	190.	10 255.4	{10 189., 10 310.}	5297.57	1.73219
33	newly 3 weeks spec	32.	1111.16	{1074., 1149.}	3372.38	1.54048	newly 3 weeks cumul	204.	4559.04	{4504., 4608.}	2134.82	1.34923	newly 5 weeks spec	18.	627.	{599., 657.}	3383.33	1.5417	newly 5 weeks cumul	235.	6018.91	{5968., 6067.}	2461.24	1.40844	newly 50 weeks cumul	268.	7850.18	{7795., 7908.}	2829.17	1.46674
34	newly 3 weeks spec	218.	2798.71	{2727., 2868.}	1183.81	1.10842	newly 3 weeks cumul	1079.	9716.74	{9629., 9803.}	800.532	0.954489	newly 5 weeks spec	118.	2028.09	{1975., 2077.}	1618.72	1.23512	newly 5 weeks cumul	1346.	14 133.5	{14 020., 14 229.}	950.035	1.0212	newly 42 weeks cumul	1517.	24 077.6	{23 781., 24 361.}	1487.18	1.20061
35	newly 3 weeks spec	0.1	1624.89	{1582., 1665.}	1.62479×10^{6}	4.21073	newly 3 weeks cumul	24.	6605.9	{6545., 6676.}	27 424.6	2.43971	newly 5 weeks spec	0.1	920.89	{882., 961.}	920 790.	3.96399	newly 5 weeks cumul	24.	8747.63	{8695., 8801.}	36 348.5	2.56167	newly 51 weeks cumul	24.	11 483.2	{11 420., 11 538.}	47 746.5	2.67985
36	newly 3 weeks spec	53.	1501.15	{1461., 1542.}	2732.36	1.45203	newly 3 weeks cumul	336.	6093.16	{6027., 6155.}	1713.44	1.25849	newly 5 weeks spec	17.	854.57	{815., 890.}	4926.88	1.70107	newly 5 weeks cumul	377.	8082.48	{8021., 8143.}	2043.89	1.3312	newly 49 weeks cumul	391.	10 634.1	{10 572., 10 699.}	2619.71	1.43452
37	newly 3 weeks spec	3.	1343.18	{1303., 1388.}	44 672.7	2.65087	newly 3 weeks cumul	35.	5477.46	{5407., 5534.}	15 549.9	2.19449	newly 5 weeks spec	0.1	753.2	{719., 791.}	753 100.	3.87661	newly 5 weeks cumul	35.	7243.8	{7180., 7302.}	20 596.6	2.31589	newly 51 weeks cumul	35.	9493.29	{9426., 9557.}	27 023.7	2.43334
38	newly 3 weeks spec	40.	1167.67	{1129., 1209.}	2819.18	1.46512	newly 3 weeks cumul	256.	4777.22	{4718., 4829.}	1766.1	1.27092	newly 5 weeks spec	12.	650.2	{619., 682.}	5318.33	1.73357	newly 5 weeks cumul	282.	6301.31	{6247., 6357.}	2134.51	1.34917	newly 49 weeks cumul	298.	8219.37	{8165., 8284.}	2658.18	1.44062
39	newly 3 weeks spec	150.	1033.22	{979., 1073.}	588.813	0.837851	newly 3 weeks cumul	734.	4241.02	{4161., 4319.}	477.796	0.761731	newly 5 weeks spec	40.	575.59	{537., 616.}	1338.98	1.15736	newly 5 weeks cumul	880.	5582.68	{5482., 5701.}	534.395	0.802307	newly 46 weeks cumul	975.	7222.73	{7040., 7419.}	640.793	0.8696
40	newly 3 weeks spec	92.	1224.13	{1188., 1264.}	1230.58	1.12388	newly 3 weeks cumul	452.	5084.61	{5011., 5150.}	1024.91	1.05109	newly 5 weeks spec	39.	673.56	{637., 706.}	1627.08	1.23695	newly 5 weeks cumul	559.	6670.37	{6588., 6750.}	1093.27	1.07672	newly 49 weeks cumul	609.	8554.11	{8411., 8679.}	1304.62	1.14752
41	newly 3 weeks spec	14.	1269.75	{1223., 1312.}	8969.64	1.95744	newly 3 weeks cumul	127.	5198.22	{5136., 5252.}	3993.09	1.61203	newly 5 weeks spec	4.	713.8	{678., 748.}	17 745.	2.2512	newly 5 weeks cumul	135.	6861.31	{6803., 6913.}	4982.45	1.70606	newly 51 weeks cumul	136.	8966.18	{8911., 9030.}	6492.78	1.81906
42	newly 3 weeks spec	202.	2068.28	{2014., 2125.}	923.901	1.01017	newly 3 weeks cumul	987.	7659.79	{7564., 7745.}	676.068	0.889882	newly 5 weeks spec	95.	1357.44	{1306., 1416.}	1328.88	1.15479	newly 5 weeks cumul	1233.	10 696.4	{10 596., 10 797.}	767.507	0.938261	newly 44 weeks cumul	1344.	16 140.4	{15 960., 16 307.}	1100.92	1.0795
43	newly 3 weeks spec	252.	1839.68	{1786., 1890.}	630.032	0.863231	newly 3 weeks cumul	1153.	6868.68	{6775., 6952.}	495.722	0.775025	newly 5 weeks spec	99.	1198.52	{1154., 1246.}	1110.63	1.08279	newly 5 weeks cumul	1387.	9561.17	{9474., 9673.}	589.342	0.838422	newly 43 weeks cumul	1536.	14 276.3	{14 075., 14 454.}	829.444	0.968203
44	newly 3 weeks spec	39.	1480.9	{1432., 1521.}	3697.18	1.57935	newly 3 weeks cumul	267.	6021.39	{5958., 6094.}	2155.2	1.35317	newly 5 weeks spec	9.	835.56	{800., 874.}	9184.	1.96742	newly 5 weeks cumul	301.	7963.44	{7906., 8016.}	2545.66	1.42253	newly 50 weeks cumul	304.	10 461.4	{10 390., 10 516.}	3341.25	1.53671
45	newly 3 weeks spec	130.	1351.24	{1308., 1409.}	939.415	1.01661	newly 3 weeks cumul	816.	5448.43	{5370., 5519.}	567.7	0.824555	newly 5 weeks spec	64.	776.64	{739., 819.}	1113.5	1.08369	newly 5 weeks cumul	987.	7257.97	{7170., 7375.}	635.357	0.866474	newly 48 weeks cumul	1048.	9661.03	{9559., 9810.}	821.854	0.964637
46	newly 3 weeks spec	72.	2274.58	{2197., 2334.}	3059.14	1.49944	newly 3 weeks cumul	478.	8728.53	{8642., 8823.}	1726.05	1.2615	newly 5 weeks spec	30.	1398.58	{1346., 1447.}	4561.93	1.66834	newly 5 weeks cumul	550.	11 913.2	{11 806., 12 013.}	2066.04	1.33566	newly 47 weeks cumul	566.	16 652.9	{16 432., 16 826.}	2842.2	1.46865
47	newly 3 weeks spec	463.	1358.95	{1287., 1428.}	193.51	0.467259	newly 3 weeks cumul	1902.	5165.56	{4988., 5408.}	171.586	0.433692	newly 5 weeks spec	209.	856.59	{806., 929.}	309.852	0.611934	newly 5 weeks cumul	2424.	7103.36	{6875., 7458.}	193.043	0.466686	newly 43 weeks cumul	2941.	10 311.3	{9930., 10 972.}	250.606	0.544484
48	newly 3 weeks spec	166.	1328.65	{1281., 1375.}	700.392	0.903121	newly 3 weeks cumul	968.	5287.64	{5187., 5363.}	446.244	0.737347	newly 5 weeks spec	51.	776.25	{744., 823.}	1422.06	1.18208	newly 5 weeks cumul	1120.	7084.2	{6958., 7184.}	532.518	0.801036	newly 48 weeks cumul	1215.	9575.42	{9373., 9779.}	688.1	0.89653
49	newly 3 weeks spec	3.	1679.51	{1624., 1717.}	55 883.7	2.74796	newly 3 weeks cumul	31.	6833.83	{6780., 6901.}	21 944.6	2.34329	newly 5 weeks spec	0.1	955.3	{915., 997.}	955 200.	3.9799	newly 5 weeks cumul	32.	9053.12	{8995., 9102.}	28 191.	2.45164	newly 52 weeks cumul	32.	11 859.1	{11 790., 11 931.}	36 959.8	2.5689
50	newly 3 weeks spec	18.	1296.52	{1249., 1343.}	7102.89	1.85736	newly 3 weeks cumul	46.	5314.46	{5246., 5372.}	11 453.2	2.06268	newly 5 weeks spec	1.	729.78	{688., 768.}	72 878.	2.86287	newly 5 weeks cumul	52.	7021.12	{6971., 7069.}	13 402.2	2.1304	newly 51 weeks cumul	52.	9178.22	{9110., 9239.}	17 550.4	2.24675
